# “Struck down by cancer with no old life to fall back on” a clinical study of illness experiences among Norwegian adolescent and young adult cancer survivors investigating the ethical implications of their illness narratives

**DOI:** 10.1002/cnr2.1765

**Published:** 2022-12-09

**Authors:** Kristine Hjulstad, Hilde Bondevik, Marit Helene Hem, Per Nortvedt

**Affiliations:** ^1^ Notodden Municipality, University of Oslo Oslo Norway; ^2^ Department of Interdisciplinary Health Sciences University of Oslo Oslo Norway; ^3^ VID Specialized University Oslo Norway; ^4^ Centre for Medical Ethics, University of Oslo Oslo Norway

**Keywords:** adolescence, cancer, care, ethics, narratives, survivorship

## Abstract

**Background:**

Cancer is a leading cause of death among people 15–24 years of age. Increasing numbers of cancer patients survive. Extensive cancer therapy may cause wide‐ranging somatic and psychosocial challenges in the lives of the survivors.

Research indicates adolescent and young adult cancer (AYA) survivors need to be seen as a distinctive group of survivors having unique health care needs. The existing literature suggests the need for specific follow‐up care programs addressing the challenges of AYAs and providing them access to specialized after care, as well as a need to explore AYA cancer survivors' own illness experiences.

**Aims:**

Through the theoretical lens of narrative medicine and care ethics, our purpose was to investigate the particular challenges encountered by the AYA cancer survivors, and how they view themselves in light of their illness experiences. We ask how AYA cancer survivors are met and understood by their medical professionals?

**Methods:**

This study applied a qualitative method using a narrative research design by collecting self‐stories of illness in order to conceptualize human experiences of illness among AYA cancer survivors. Eight in‐depth interviews were conducted using a narrative analysis according to the narrative plots of *restitution*, *chaos* and *quest,* as suggested by Arthur Frank.

**Results:**

Hopelessness and a struggle to take part in the activities of daily life as survivors of cancer were revealed. Too ill to fully take part in the society, the AYA cancer survivors strive to be understood for what they are, namely young survivors.

**Conclusion:**

The survivors'stories reveal a moral imperative that needs to be honored by medical professionals in order to improve cancer care. Cancer survivorship may be a lifelong process necessitating long‐term follow‐up care. With the lack of specific care programs for AYA cancer survivors, follow‐up care is provided by general practitioners or other medical professionals, who often lack expertise in the unique challenges faced by AYA survivors. Because they feel their needs are unmet and their stories not understood, the AYA survivors might experience a sense of abandonment. By adopting a care ethics and narrative medicine approach we provide medical professionals a theoretical framework to better understand and care for AYA cancer survivors. Clinical trial number is 2012/1141.

## 1 BACKGROUND

Cancer survival rates have increased over the past 40 years due to modern cancer therapy.^1^ Still, the incidence of cancer in adolescence is increasing^2^, ^3^ and cancer is the leading cause of disease related deaths in people aged 15–24.^4^, ^5^ Numbers show malignancy, followed by accidents and suicide, are the most common causes of death among adolescents and young adults.^4^ Survivors of cancer in adolescence and young adulthood (AYA‐cancer survivors) are commonly in need of specific services following cancer therapy as they are at risk of developing long‐term effects from treatment that can influence their psychosocial and physical functioning.^2^ Although research show many AYA cancer survivors are satisfied with their cancer care a great number of AYA cancer survivors report unmet medical needs a long time after termination of therapy.^2^ The reason why some AYA cancer survivors are satisfied with their cancer care whilst others are not, is not fully understood and may indicate a need for further research. Hopefully this study contributes to a broader understanding of the reasons why there are unmet needs of care reported by AYA cancer survivors. As the results of this particular study reveal there are great differences in the cancer care of AYA cancer survivors between countries.^6^ A recent meta‐ethnographic study claims our understanding of cancer survival among young adults is limited in terms of medical concerns, lived experiences and social and cultural phenomena.^7^ It further claims there is a need for research that help to expand our knowledge of the ways in which “young adult cancer survivors are understood, understand themselves, and narrate their lives”.^7^ Another recent study claims that while the AYA cancer survivors have unique health care needs, the follow up care is not tailored to meet their particular challenges and there is therefore a need to explore AYA cancer survivors' own experiences.^8^ A cancer diagnosis during adolescence or young adulthood generates unique medical and psychosocial needs because developmental milestones are simultaneously impacted.^9^ A Norwegian research article on adolescent survivorship of sarcoma describes the survivors as having reduced social lives due to their decreased functionality. Young survivors experience specific challenges when it comes to education, work, social life, identity and having a family.^10^ The lives of AYA cancer survivors are, according to another Norwegian study, profoundly changed not only while undergoing treatment but also over time as a result of the diagnosis.^11^ A Danish article claims that AYA cancer survivors are at risk of becoming marginalized and individualized, making them all the more vulnerable unless their particular illness experiences are investigated.^12^ Thus, AYA survivors need close follow up, screening and intervention throughout the entire trajectory of cancer.^11^

There is a lack of consensus as to what age defines adolescents and young adults (AYA).^5^ Internationally the age range is seen to be 15–39 years.^2^ In Norway, the group of AYA cancer survivors is defined as 15–35 years of age. According to the Norwegian AYA cancer foundation, UNGKreft, in Norway this age range is not specific to diagnosis. The wide age range is meant to include young patients at a particularly vulnerable stage in life as they try to establish themselves as adults whilst developmental milestones are significantly influenced by their diseases. Their challenges include educational challenges, career choices, psychosocial issues, infertility problems and difficulties with body‐image and feeling alienated from their peers as a result. UNGKreft is now considering expanding their age range to include people up to 39 years old.^13^

Internationally, AYA cancer patients are gradually being recognized as a distinctive population in oncology as a result of the unique challenges this group faces.^6^ Still, cancer therapy is commonly provided to young patients across pediatric and adult cancer services.^5^ Neither category seems appropriate for addressing the specific needs that are salient to the health of the AYA patients. Unmet care needs among the AYA population may be related to the traditional dichotomy of pediatric and adult oncology services.^6^ Although some countries like Canada have AYA survivorship care programs^6^ no such programs exist in Norway. According to UNGkreft, follow‐up care after termination of cancer therapy is mostly provided by general practitioners. Regarding psychosocial, financial and educational challenges, the AYA survivors heavily depend on peer work from the various cancer foundations.^13^ The lasting physical and psychosocial late effects of cancer in AYAs are only understood by health‐care professionals to a limited degree.^6^ If we are to understand the impact of cancer on the lives of AYA cancer survivors, then they must not only be seen as a distinctive group of survivors, but also offered better follow up care, including more late effect clinics.^3^ Health‐care professionals need to be upskilled with specific competence in caring for and communicating with AYA cancer survivors and further must be sensitive to their needs and validate their different experiences.^11^

Survivors of cancer attempt to transition to a “new” normal life.^6^ The problem of transitioning from ill to well is described by Susan Sontag as holding dual citizenships, in the kingdom of the well and in the kingdom of the sick.^14^ The Canadian sociologist Arthur Frank refers to this dual existential state as a remission society whose members are effectively well but cannot consider themselves fully cured.^15^ The term “remission society” serves to describe a sense of homelessness felt by the chronically ill who are neither ill, nor well, moving continuously between the kingdom of the sick and the kingdom of the well, as their illness moves in and out of the foreground. The conflict between sickness and wellness is not unique to any specific age group, yet it goes to show that successful cancer survivorship is not limited to surviving cancer alone, but includes the quality of a person's life following cancer therapy. To AYA cancer survivors these issues seem even more pressing. This study explored the transitions AYAs make between ill and well whilst moving from childhood to adulthood by investigating their stories of self as illness narratives.

### 1.1 Illness narratives

By telling one's story, one can give meaning to the seemingly meaningless. The AYA cancer survivors in this study were just about to establish a life of their own as adults when interrupted by disease. This seemingly senseless situation might be given a sense of meaning through the act of telling their stories to others. Narrative medicine is, according to one of its founders, Rita Charon, “medicine practiced with the competence to recognize, absorb, interpret and be moved by the stories of illness”.^16^ Narrative is an instrument of self‐knowledge and a way of making meaning and “coping with the contingencies of moral and mortal life.”^16^ Approaching the patients with narrative competence allow us to recognize how patients' illness experiences are emphasized by their stories.^17^

As our aim was to investigate the particular challenges encountered by the AYA cancer survivors, and how they viewed themselves in light of their illness experiences, we studied their stories of the self through a narrative lens. In these narratives, the teller is the AYA cancer survivor while the listener is the medical professional caring for them. By telling their stories, the AYA cancer survivors have chosen a certain narrative identity for themselves. These identities were investigated in this study by using the plots suggested by Arthur Frank.^15^ Narrative competence allows health care professionals fathom what their patients are going through and better grasp their experiences.^17^

To the teller, the story is the bridge between their past self and their present self. A Canadian study investigated how illness narratives served to renegotiate identity among a heterogeneous group of cancer patients. It found that cancer survivors' illness narratives helped them negotiate their way through treatment regimens, changes to their bodies and disrupted lives. Thus, their stories became vehicles for making sense, not of an illness, but of a life.^18^ There has been a great progress in the scientific understanding of cancer in adolescents and young adults. Still, several research gaps remain and our understanding of young cancer survivors' illness experiences is limited.^2^, ^7^ Thus, there is a need to explore their illness narratives further. The illness experiences of young adult cancer survivors resemble those of the chronically ill. Experiences of surviving cancer while young have no expiration date. Accordingly, new perspectives on and interpretations of these experiences are essential.^7^ It is our hope that this study contributes to establishing new perspectives.

### 1.2 Ethical and philosophical perspectives

To capture the central values inherent in the participants' narratives, we focused on participants' subjective experiences and personal narratives, as well as their relationships with family, friends, and medical professionals. The ethics of care is the ethical perspective most relevant to this study.^19^ Care ethics builds on the notion of caring for the dependent and vulnerable. It is more of a practice or virtue rather than a theory and thus implies there is “moral significance in the fundamental elements of relationships and dependencies in human life”.^20^ Traditional moral philosophy focuses on the role of judgments and how universal moral principles can guide agency, and thus tends to overlook the important role of moral perception and of emotions in moral life.^21^, ^22^ In contrast to this “blindness” of universal and impartial moral theories, the focus on the perspective of care in moral philosophy in general, and in medical ethics and nursing ethics in particular, better captures the role of emotions and moral perception in moral agency.^23^, ^24^, ^25^ Attentiveness, the ability to understand the particularities of situations and the subjectivity of persons, is of crucial importance when encountering the illness experiences of these young adults.

## 2 AIM AND RESEARCH QUESTIONS

Surviving cancer successfully entails more than just immediate physical survival. It has been well established that AYA cancer survivors have a higher risk of long‐term and late effects of cancer, and they are in a unique phase of life in regard to psychosocial, cognitive and emotional transitions.^2^, ^6^ Still, the narrated experiences of young adult cancer survivors have not been well studied, and we therefore aimed to develop new insights into this topic through our investigation.

This study attempted to establish a better understanding of what it is like to make the transition from being sick to being well, struggling with side effects of cancer, while also transitioning from childhood to adulthood. Conducting a narrative analysis, allowed us to arrive at a broader understanding of how the AYA cancer survivors talk about their illness experiences and what truths they reveal about themselves. The aim was to explore how illness stories help survivors negotiate a new identity based on their illness experiences. Moreover, we explored the ethical implications inherent in these stories to health care professionals in general and general practitioners in particular. The following questions were addressed:

- What particular challenges do AYA cancer survivors have?
- How do they view themselves in light of their illness experiences?
- To what extent do AYA cancer survivors feel their concerns are addressed and understood by their medical caregivers?

## 3 METHODS

### 3.1 Design

This study applied a qualitative method using a narrative research design that involved collecting self‐stories of illness to conceptualize human experiences of illness among AYA cancer survivors. In our analysis, the narrative plots of “restitution,” “chaos,” and “quest” as suggested by Arthur Frank (see below) were adapted to the narrated experiences of the participants.^15^ This design allowed us to develop a new and deeper understanding of groups of patients with very diverse and complex illness experiences.

### 3.2 Participants

Participants were recruited via the Norwegian Cancer Society by attending patient association meetings, as well as via Facebook and posters in oncology clinics. Adolescent survivors of cancer who had diagnosed with cancer or had had a recurrence in the age‐range of 15–35 years and at least 1 year post‐treatment were invited to participate. Each potential participant was made aware of the study procedures, risks and benefits. Written consent was obtained prior to the interviews and all participant identifiers were removed and the participants were given fictitious names. Eleven people wished to participate. Two participants declined to join after the initial contact was made. One had a relapse and was receiving therapy and therefore no longer met the inclusion criteria. Eight participants remained, two of which were men. Four participants were survivors of childhood cancers, while the others had been diagnosed in their teens. At the time of the study, the participants' ages ranged from 18 to 35.

### 3.3 The interviews

Eight loosely semi‐structured, in‐depth qualitative interviews were conducted using open‐ended questions. The first questions were designed to make the participant freely tell his or her story.^26^ The other questions covered follow‐up care, side effects of therapy, social and educational obstacles and thoughts on family and the future. All interviews were tape recorded and transcribed verbatim by professional transcribers. The interviews were conducted by the first author and lasted 60–90 min. For the interview guide, see Table 1.

**TABLE 1:** Interview guide

*Main question 1: The self and experiences with cancer*
Would you please tell me about yourself and your experiences in light of having struggled with cancer at an early age?
Follow‐up topics if necessary:
What were your thoughts on cancer as a disease before you got ill yourself?
How would you describe yourself as how you were prior to your diagnosis?
*Main question 2: Social network and education*
How did friends, family and co‐workers, etc. respond to you disclaiming having had cancer or even having side‐effects of cancer therapy (if any side‐effects are mentioned)
Has your disease affected your education or career choice either positively or negatively?
Follow‐up topics if necessary:
Did your diagnosis in any way change or affect friendships/relationships to people in anyway?
If you had to receive special tuition or assistance in school as a result of the disease and therapy, then what are your experiences with this?
Have you ever experienced receiving either positive or negative attention to having gone through a cancer disease, and what do you think of the attention you received?
Have you ever felt being different to others as a result of what you have gone through?
*Main question 3: Biographical breach*
As cancer therapy ended, to what extent did you feel you could pick up where you left and return to the life you had before you received the cancer diagnosis?
Follow‐up topics if necessary:
Suffering from severe disease may be regarded as a life‐crisis and a chock, and some may have difficulties finding a way to cope with the realities of things. What are your experiences and thoughts about this? What was your way of coping?
Do you feel your self‐image has been affected by your disease either positively or negatively?
*Main question 4: Health care*
Many cancer survivors may have a greater need for health care as a result of long‐time side‐ effects of cancer therapy.
If this applies to you, to what extent do you feel understood and your concerns met by your general practitioner?
Follow‐up topic if necessary:
What are your experiences with communicating your concerns and medical problems to your physicians during and after cancer therapy?
What advice with you give health care professionals that are working with cancer survivors?
*Closing questions*
Would you like to listen through the recording?
Is there anything you wish deleted?
Are there any questions I should have asked you, or not have asked at all?

### 3.4 Data processing

The transcribed interviews were read closely by three of the authors (KH, PN, and MHH) individually to identify emerging themes, topics and plots. The authors met regularly to compare and discuss their individual findings. The fourth writer (HB) was involved after the initial analysis was done to secure validity by undertaking an independent analysis confirming the identified themes and plots were reflective of the data.

### 3.5 The analysis

A narrative analysis was conducted looking at the themes that emerged during the interviews as well as how the tellers structured their stories of self. The themes and structures are discussed separately. Firstly, themes and topics of concern to the participants were identified and considered together to develop larger groupings of themes and categories. Second, we re‐read participants' stories to determine how they were constructed, meaning we looked not only at the contents of the narrative but also its form (i.e., temporal course, plots, and subplots) in order to achieve insight beyond the spoken words.^16^ The co‐authors read and discussed the transcripts several times to develop ideas across interviews. Finally, they re‐read the transcripts to look for narrative structures to understand how the participants constructed their stories and their narrative identities with special focus on temporality and plot.

The structural part of the analysis was largely inspired by Arthur Franks book *The Wounded Storyteller*, and attempted to see if the participants' stories could be understood according to his typology of narrative plots. A plot is defined as the components that give a story meaning or establish meaningful causal relations between events.^16^ Frank suggests three plots: the restitution plot, chaos plot and the quest plot.^15^

- Restitution

The restitution plot reflects the desire to recover from illness and has become the narrative that models how illness is to be described.^27^ Restitution stories are about the triumph of medicine.

- Chaos

The chaos plot imagines life never getting any better.^28^ Even when the chaos of diagnosis and fighting for survival has subsided, the survivors still experience a lot of emotional battering. Fighting the side effects of cancer may represent a loss of control; hence, the chaos plot moves into the foreground. As chaos continues to be a part of the survivors' story, they continue having trouble remaking a sense of purpose.^28^

- Quest

The quest plot is defined as the survivor having accepted his illness and believes something is to be gained through the experience. These storytellers accept illness and seek to use it. There is a sense of purpose, and the survivor may desire to help others.^29^

### 3.6 Ethical concerns

As the quality of the data collected could potentially activate a reflexive process among the participants and lead to psychological distress, the participants were offered support by a nurse representing the Norwegian Cancer Society. The study was reviewed and accepted by the regional ethics review board (REK in Norway). File number 2012/1141.

## 4 RESULTS

### 4.1 Sample characteristics

Two men and six women aged 21–34 participated in this study. They had been diagnosed with different cancers at different stages of late childhood and early adolescence. One participant had ovarian cancer. Both men suffered from tumors of the central nervous system (CNS) and four women suffered from acute leukemia. Only one of the men had a higher education and a steady job. Four of the six women were pursuing a higher education while one had a steady job. All participants suffered some degree of late effects of cancer and felt milestones in adolescence had been significantly affected by their disease. Four participants were single and claimed this was a result of their disease. The other four were in partnerships. Neither of the male participants was in romantic relationships. The sample characteristics are based on the information spontaneously provided during the interviews as no one was asked specifically about marital status or other personal details. See Table 2.

**TABLE 2:** Sample characteristics (*N* = 8)

The age‐range of the participants upon diagnosis	10–23 years
The age‐range of the participants at the time of interview	21–23 years
*Gender*	
Male	2
Female	6
*Diagnosis*	
Brain/CNS tumor	3
Acute lymphoblastic leukemia (ALL)	2
Acute myeloid leukemia (AML)	1
Lymphoma	1
Ovarian cancer	1
*Marital status*	
Single	4
Married/partnership	4
*Family status*	
Have children	2
No children	6
*Employment status*	
Full time	1
Part time	2
Student	4
Social assistance	1
*Education*	
Junior high	1
High school	2
College degree	4
Graduate degree	1

### 4.2 Three emerging themes

Several themes emerged from the interviews. All participants addressed three major themes, which are the basis for this study. The first theme deals with the situation of being neither ill, nor well because of the struggle with the side effects of cancer therapy. The second describes the complicated life phase in which AYA cancer survivors find themselves as they transition from childhood to early adulthood while battling these side effects. The third theme comprises the sense of abandonment and continuous chaos the participants experience when they have no one to turn to for help with their troubled bodies and troubled selves.

#### 4.2.1 Neither ill, nor well: A remission society

The experience of being neither ill, nor effectively well was explicitly described by all of the participants: “My life is split in two. I live two different lives, and it takes a lot of hard work to separate the two*”*. Anita (27) is a survivor of acute lymphocytic leukemia (ALL). She was diagnosed at the age of 13. Her story reveals a constant struggle to balance two selves: one as the cured cancer patient and the other as a person fighting long term side effects of cancer therapy.

Margret, a 30‐year‐old anthropologist, got sick while at the university: “Once you have finished therapy you think 'Hurray, I am healthy! '. But in reality, you are young and cured, but still you have a body that has been beaten up badly for months and no longer works as it should.”

Another woman, a 22‐year‐old nursing student named Sophie, described the frustrations of facing side effects after cancer therapy while the people around her expected everything to be back to normal very well: “People expect you to be all good when you finish cancer therapy. But what actually happens is that one thing after another shows up, and maybe you will never be any better. I ended up with osteoporosis and a prosthetic hip, and then there is this extreme tiredness and I couldn't even go to school”. She added: “You hardly hear about side effects. You're not prepared. It comes like a shock. You are treated to be cured, but you kind of don't get well. And you certainly cannot start living just like your friends do when you are out of it.”

To some, remission from cancer is even more devastating than the illness itself: “Cancer therapy is like entering a machine only to get out of it with a weaker body and fewer options in life,” said Jack, a 21‐year‐old man, who had survived multiple brain tumors at the age of ten and during his early teens. He never managed to finish school or get a higher education. He was still struggling with finding a job and making friends.

As survivors of cancer, all the participants expressed frustrations related to the many severe side effects they suffered after cancer therapy. They felt unprepared for the situation they found themselves in and further expressed a sense of hopelessness at not being effectively well despite being cured from cancer.

#### 4.2.2 In a unique position: Transitions from sickness to wellness, and from childhood to adulthood

The conflict between sickness and wellness coincides with the AYA cancer survivors' young age at the time of diagnosis, which puts them in a unique position. As Anita explained, “It is a special thing getting sick as a teenager. I had not had the chance yet to develop a life as an adult.”

June (27), who had been diagnosed with ovarian cancer at the age of 16, was struggling with chronic fatigue syndrome (CFS) and lung fibrosis: “I went back to school after I got sick. That became very difficult. I was so terribly tired. I did not manage to take part in the class. Besides, I had completely different experiences and a completely different perspective on life than my class mates. My class mates were not any nice to me at all. Because I had been away from school so much, I was no longer welcome in the group. I have to live life completely differently from how I did before.”

Margret described it as a shocking transition when her cancer therapy was over: “I really had nothing to go back to. I mean I had a sick leave from my studies, but no job. All the friends I had had something to do. I just had to sit there with nothing. You know, cancer is not just like an interruption or a break from your life the time you are in therapy and then when it's over you can just pick up where you left off!”

Ingrid (34), who had twice survived a brain tumor, felt as if she had lost important years of her life to cancer. “Ten years really, yes definitely 10 important years from the time I was 20 till I was 30, have been stolen from me, sort of. I feel I lag a bit behind other people at my age.”

Caroline (20) had to give up law school when she was diagnosed with leukemia. “Getting cancer this young is a very special thing. You are neither a child nor an adult, and yet in a difficult situation like that (having cancer) you are supposed to figure out who you are!”

In these examples, the participants describe a sense of being cut off from the lives they used to live. They are standing on the threshold of adult life, struggling to navigate a new life after cancer while feeling they have lost important years of their adolescence to their illness. Their experiences are so different from those of their peers that they sense a kind of alienation. Restitution of normality after undergoing cancer therapy is that ideal; however, all the participants seemed to find it difficult, apart from one, Mark, making him an exception to the rule.

Mark (30) was diagnosed with a CNS tumor. This caused him to end up paralyzed, impotent, and infertile and with two ostomy bags. “Despite all that, I have a life pretty much like the one I had before I got cancer. I still get to do the things I love. I have been very lucky to have a close network of friends and family. I have also been very fortunate to have an employer who said, the minute he found out about my cancer, that my job was the last thing to be worried about.” Mark's story indicates the importance of a well‐established network of friends, family and colleagues for being able to pick up where one left and move on—in other words, having an old life to fall back upon.

#### 4.2.3 Hopelessness and no places to turn for help

So far, our findings reveal the hopelessness of adolescents who are neither being sick nor well after battling cancer therapy. Their sense of hopelessness, it seems, is even greater when there are few places to turn for help. All the participants expressed frustration at having nowhere to turn for follow‐up care, or having to re‐tell their stories every time they see a new health‐care professional or social worker. Three of them were more articulate than the others, putting into words the frustration that all participants had to some degree.

For Ingrid, the trouble started when she was discharged from the hospital. She struggled greatly with both the side effects of cancer therapy and not finding a doctor to provide her with the follow‐up care she thought she needed. Secondly, friends and family expected everything to be back to normal when her cancer treatment ended. However, Ingrid suffered from fatigue and depressions and felt left to herself when her parents said she should be happy and just pull herself together. Ingrid was also without a job and as she tried to explain her situation to her social workers, she felt they, too, were unable to understand the limitations of her work capacity: “Nobody understood what I felt. It feels horrible when neither nurses, doctors, nor social care takers seem to understand.”

Mark claimed that a greater competence in understanding each patient's unique challenges is required: “Many of us get sick while we still are kids in the children's ward but end up being transferred to the cancer ward for adults. It is very important to make that transition smooth. This is not just about the hospital as a system: every individual medical professional must keep in mind what that means to the patient.”

Ingrid said, “I really wish someone had helped me or at least given me some advice to make the transition from sick to well a little better – helped me return to a normal life. I just felt incredibly empty after the treatment”.

Mark expressed a similar concern: “Being an adolescent in a situation like that, is completely different from being an adult. We are just starting our lives! I think the medical professionals need to understand that we are not like other adolescents. The doctors should think of what we, the young patients, are concerned with and adjust the way they think and talk to us!”

Caroline sometimes felt she was not taken seriously by the medical professionals treating her: “I was constantly compared to the older patients. Therefore, I felt they didn't take my concerns seriously.”

The participants claimed they were not offered follow‐up cancer aftercare that adequately addressed their specific needs. This, it seems, contributed to a greater sense of hopelessness when battling the side effects of cancer and feeling neither ill nor well. The participants also seemed to hold the medical professionals responsible on a more personal and individual level rather than seeing this is a problem related to how medical care is structured and organized.

### 4.3 The survivor as narrator – narrative structures

Because this study was interested in how the participants revealed their selves through the act of telling their stories, we shifted our attention from what was being told to the telling. This shift meant looking at how the stories were organized and put together to better see what a particular story did and really was about. Attention to narrative form adds insight beyond what a simple focus on themes can provide alone.^30^ Therefore we took a structural approach to narrative analysis, focusing on the plots found in the participants' stories of self. Through the act of telling, the participants chose a narrative identity for themselves. We investigated this mechanism further by using the narrative plots suggested by Arthur Frank as listening devices.^27^ Below we describe only the stories of the participants that best illustrate Frank's plots, although all participants' stories were analyzed according to Frank's plots.

#### 4.3.1 A story of restitution

Sophie's story is one of restitution. Her restitution is not limited to the absence of pain, but also includes the establishment of new meaning in her life. She has gained a reflective grasp of what she has lived through. The choice of restitution rather than chaos as the dominant narrative element relates to her story's turning point: “It probably was what my doctor told me: “There is a chance you never will get any better, but if you do, it is a bonus.” Well living with that uncertainty—will I get any better or not?—that's frustrating. But after that, I started thinking this is as good as it gets. This is all I can expect. I was no longer waiting and hoping for something better. It was impossible to find peace that way.”

Being a cancer survivor requires accepting some level of illness. Sophie has accepted her illness after having received help in doing so: “I started getting help from the clinic for chronic pain. They have a different way of thinking and know what I am going through.”

#### 4.3.2 A story of chaos

Chaos dominates Ingrid's story: “I wanted to give up. But the reason why I am disabled today is not the cancer. It is that I have not received any help. That is why I am disabled. It is no fun in receiving unemployment benefits. I feel I am staying put. This happened when I was 20, now I am 34, I still haven't recovered. I should have been working. I don't believe in myself anymore. Nobody needs me.”

In Ingrid's case, the restitution narrative fails. And for the chronically ill there are no other stories to fall back upon. Their sense of chaos and hopelessness continues and must therefore, be acknowledged by medical professionals and other care takers. This need is both moral and clinical, as Frank puts it.^28^

#### 4.3.3 The quest

The teller of a quest story has gained an insight that must be passed on to others.^29^ Mark's story is a quest story: “My dream is to somehow influence people's lives – to make a difference. So, I am an active member of the patient's associations and assist others in a similar situation.”

All participants were involved in the patient associations to varying degrees believing they should pass on the experiences they had gained. As Mark put it, “It is about using the negative experiences, and turning them into something positive.” Mark used this logic to explain why he visited with newly diagnosed teens in the hospital wards, saying: “I feel I have something to give to others.”

Although he is constantly being interrupted by illness, Mark is grateful for whom he has become. Even if he must be in a wheelchair, he is grateful for the perspectives on life he has gained: “What matters is to make a difference in somebody's life.”

#### 4.3.4 Multiple stories, one narrator

The participants' stories had different plots depending on what they were talking about and the situation they were describing. In illness narratives, all the suggested plots appear alternatively and repeatedly. Still, at any given moment, there is one plot that predominates. Accordingly, the participants revealed the coexistence of multiple plots, thus serving as an example of the fact every storyteller is telling multiple stories.^27^

Though Mark has an explicit quest, his story is just as much a story of restitution. It illustrates the importance of having an old life to fall back upon as he had completed an education and even had a job prior to his diagnosis.

In some of the stories, the narratives of chaos and restitution coexist and fight each other. Anita lives a relatively normal life, still feels the need to hide the side effects of her treatment as though she has something to prove, namely that she is still the same person. While she tells herself that everything will be as it was before, that she is “a regular person and no longer a cancer survivor”, she is frustrated by the appearance of new side effects. “I realize I have no idea what the future brings,” she says, thereby admitting she will likely forever be a member of the remission society and continue to be neither sick, nor well.

## 5 DISCUSSION

### 5.1 Interruptions

Living with illness is living with perpetual interruption.^15^ This study revealed how chaos still interrupts the participants' stories when they experience new side effects of their cancer therapies. For AYA cancer survivors, chaos will always remain their story's background and occasionally reappear in the foreground.^27^ For those who truly have reached a state of restitution, health is no longer about the absence of illness but a new way of coping and living. This allows for a new theoretical approach to understanding young cancer survivorship—an approach combining a narrative understanding of the patients' stories of the self and the perspective of medical ethics.

### 5.2 A new approach

AYA cancer survivors face significant challenges when transitioning into adulthood. For one, they may become marginalized as patients are medically and legally considered children until the age of 16 years. Still, the challenges facing the AYA cancer survivors are far different from both pediatric and adult cancer survivors.

A growing number of research articles examine the various aspects of the experiences of young adult cancer survivors, but still this evidence suggests a strong need for an expansion of the knowledge of how AYA cancer survivors are understood and understand themselves.^7^ Cancer in adolescence and young adulthood is associated with a more negative life disruption than is seen in older adults and adjusting to survivorship may be a lifelong process.^11^ Despite extensive research on survivorship in adult and pediatric patients, few studies report outcomes specific to AYA cancer survivorship.^6^ Cancer in adolescence and young adulthood may cause substantial disruptions in schooling and career progress, in addition to creating challenges in resuming daily life activities.^2^, ^6^ Knowledge about the specificities of the challenges this group faces must reach the medical professionals who are to care for these survivors in a clinical context, as there appear to be a lack of sufficient knowledge specifically targeting the needs of AYA cancer survivors. Some countries have developed dedicated AYA cancer programs and AYA oncology has emerged as a discipline internationally. Nevertheless, there are still considerable differences between countries in terms of the support they offer.^6^ In Norway, no such targeted programs exist and internationally there is still no optimal survivorship care model for AYA cancer survivors.^6^, ^13^ As a result, AYA cancer survivors often struggle to access adequate health care during early life transitions. This may in turn negatively impact their health.^2^ It is of great importance to view all these negative outcomes as connected to one another as clusters of symptoms and late effects of cancer as well as predictors of impaired function.^11^ As AYA cancer survivors end their cancer therapy, contact with their health care teams will diminish. In situations where there is no dedicated follow‐up care for AYA cancer survivors, the follow‐up care will be provided by the general practitioner, as is the case in Norway.^13^ But because both general practitioners and medical professionals in hospitals only see a limited number of AYA cancer patients they are unfamiliar with their specific needs and have limited experience in AYA care.^6^

### 5.3 Young members of the remission society

The participants in this study illustrate both the difficulty of recovering from cancer and the extent to which the transition to early adulthood is affected by their illness. Though the participants have reached a “new” normal life, the restitution plot becomes problematic when the survivor is still battling the side effects of cancer therapy. Considering how the restitution plot has become the preferred narrative of institutional medicine and powerful interest groups, and has thus come to shape the culture of illness, survivors may feel there is nowhere to turn when other plots prove possible. Medicine still tends to view people as either sick or well. Arthur Frank, however, argues that being a survivor of cancer is less about holding dual citizenships than it is about living with perpetual chaos in a so‐called remission society. In a remission society, sickness and health constantly blend into each other.^15^ Suffering from the side effects of cancer, the AYA cancer survivors need to accept some level of illness as a permanent background to their lives.^15^

AYA cancer survivors are particularly vulnerable. A similar vulnerability is more poetically addressed by Remarque in his novel about World War I, *Quiet on the Western Front* (1929). The young soldiers are described as “standing on the very threshold of life itself” not yet having “had a chance to put down any roots.” The war has made them feel cut off from everything they knew to that point. Returning from war, they have no old lives to fall back on, as opposed to the older soldiers who have firm ties to their earlier lives.^31^ AYA cancer survivors can be regarded in much the same way. Cancer, its treatment, and late effects represent a break from the lives lived before the time of diagnosis as well as a loss of the opportunities that existed prior to the diagnosis.^32^ Though the participants' stories revealed a sense of restitution, chaos remains inherent in the participants' stories as they continue to be troubled by side effects of cancer. Thus, each individual story seemed to contain some elements of each unique plot, thereby suggesting that every storyteller has multiple and sometimes conflicting stories to tell.

### 5.4 A lack of care, a lack of connectedness

Though many of the frustrations the participants expressed could be connected to how the health care systems and of follow‐up care are organized in Norway, the participants expressed their frustration at having their needs unmet and revealed a sense of hopelessness when not provided with sufficient information or not feeling understood by their medical professionals

Health care professionals witness the plight of others. Health care is about bestowing attention on the patient. All clinicians act as meaning‐making vessels for the patients when they recount their individual situation.^33^ The AYA cancer survivors' subjectivity is represented by their unique and personal perspectives and their suffering. In terms of narrative medicine, the ethics inherent in this tradition regards illness stories as pivotal to the person's suffering.^33^ The health care professionals accompanying a patient through a cancer trajectory have a responsibility to hear the patient out.^33^ To flee from a patient's story is to flee from the patient, and shows a lack of care. A lack of care in a patient‐doctor relationship will cause the patient to feel abandoned in chaos.^34^ Care is not simply a matter of effective treatment and communication skills. Care is also a way of responding and acting towards another human being in a professionally appropriate and benevolent way.^34^ Care is just as much a relational competence as it is a part of clinical agency and moral life

The harm caused in relationships by a lack of care and responsibility is the main focus of the ethics of care.^34^, ^35^ To care assumes a certain sense of connectedness and dependency. The ideal of detachment and non‐interference, which may occur in some physicians' relationships with their patients, is contrary to an ethics of care and responsibility, as this ethical perspective believes that the absence of connectedness and care eventually will cause harm to the patient. There is a risk of non‐interference in a tradition of medical ethics that strongly emphasizes patient autonomy.^33^, ^34^ By not listening to the AYA cancer survivors' concerns, the medical professionals might contribute to a sense of abandonment and chaos.^36^

Many care ethicists emphasize that ethical responsibility, in this case doctors' responsibility to their patients, must fundamentally be seen as an answer to the voice of illness and the expressions of vulnerability, as well as to the strength and the resilience shown by AYA cancer survivor.^37^ This sensitivity to the experiences of patients implies seeing and being aware of the moral realities illustrated by the patients' narratives. This receptivity and capacity for moral perception is at the heart of a patient‐centered ethics, as well as an ethics of care and responsibility in health care.^19^

The lasting physical and psychosocial late effects of cancer in AYA cancer survivors are only understood by health care professionals to a limited degree.^6^ The AYA cancer survivors may therefore be receiving technically adequate care while being abandoned to deal with the consequences of illness alone.^16^, ^38^ As research has revealed, living with cancer and its treatment early in life involves profound and dynamic identity work.^39^ Health‐care professionals need to be upskilled with specific competence in caring for and communicating with AYA cancer survivors and further must be sensitive to their needs and validate the different experiences of the AYAs.^11^ An understanding of the patients' illness experiences as narratives, and as a way of revealing self, is likely to increase health care professionals' ability to care for the sick. As Rita Charon puts it, “Narrative medicine provides health care professionals with practical wisdom in comprehending what patients endure in illness and what they themselves undergo in the care of the sick.”^16^ Most survivors of AYA cancers would benefit from tailored guidance related to the physical, psychosocial and emotional challenges they may face.^32^ However, as there is no general plan for follow up care of AYA cancer survivors in Norway, one cannot expect any general practitioner or other doctor to be fully capable of providing AYA cancer survivors all the help they need. We do, however, hope this study provides the doctors coming in contact with AYA cancer survivors a template and some suggestions for how to approach the AYA survivors' sufferings.

### 5.5 Implications and limitations

This study provides further insights into how AYA cancer survivors understand themselves after going through cancer therapy while attempting to re‐enter into the lives they once lived. They are forever affected by their experiences. With its narrative approach, this study provides knew knowledge as to how the experiences of young adult survivors are narrated as individual stories of illness and survivorship. We believe this is of great importance because the experiences of the young survivors 1 day will become the experiences of survivors having grown old.^6^ The ethical imperative inherent in these stories will therefore remain. Furthermore, by adopting the approaches of narrative medicine and care ethics, this study provides clinicians a new theoretical framework for better understanding and caring for AYA cancer survivors.

One important limitation is the small number of participants. The recruitment of AYA cancer survivors willing to tell their stories is difficult. Surviving cancer is a lonely endeavor possibly still associated with stigma, which means that important voices may not have been heard. Another weakness is the gender balance as only two men wished to participate. Differences in time periods between the different stories have not been investigated due to the small number of participants. It is likely, however, that the participants told their illness stories differently and had achieved different levels of reflexivity regarding their cancer trajectories based on the length of their rehabilitation period and the time since diagnosis. The relatively low sample size may cause a risk of bias as only the stories wanting to be heard were told. Meaning, there is a chance of a “non‐response” as only those feeling strongly about the topic participated thus challenging the variability of the results. Still, qualitative studies can reach saturation at relatively low sample sizes when the objectives are narrowly defined as in this study.^40^ We believe we have gathered data to support our theory, but more work needs to be done by other researchers to lend support for our theory in studies with larger samples and higher participation rates. We believe, however, our analysis is transparent and the findings are consistent, meaning we present a valid number of experiences of cancer survivorship in AYA cancer survivors.

## 6 CONCLUSION

The results suggest a sense of hopelessness among the AYA cancer survivors. Despite being cured from cancer, they still cannot regard themselves as well. Furthermore, the findings speak to a lack of information specific to this group, as well as a lack of places available for help and follow‐up care. The existing literature stresses the importance of establishing developmentally appropriate support for AYA cancer survivors and taking a biopsychosocial view of AYAs during both cancer therapy and survivorship.^6^, ^11^ It is important to understand the ways in which AYA cancer survivors adjust to the experience and the subsequent quality of their survival is important to understand, given the many years of life that lie ahead for young people following successful cancer treatment.^5^ The core of the follow‐up care provided cancer survivors in general, and to AYA survivors in particular, is the ability to recognize the ethical appeal of the survivor's story, to listen to and acknowledge their plight, as well as to act in accordance with an ethics of care. The imperative to do so cannot be reduced to a matter of having enough time; rather, it is a way of seeing people, of being present and sensitive to the other. A narrative oriented ethics, based on narrative techniques, may help the individual medical professionals to acquire new tools and communication skills that may be useful in their clinical encounters with young cancer survivors.^17^

## AUTHOR CONTRIBUTIONS

**Kristine Hjulstad:** Conceptualization (equal); formal analysis (lead); methodology (lead); project administration (equal); writing – original draft (lead). **Hilde Bondevik:** Methodology (equal); supervision (equal); writing – review and editing (equal). **Marit Helene Hem:** Formal analysis (supporting); methodology (supporting); supervision (supporting); writing – review and editing (supporting). **Per Nortvedt:** Conceptualization (equal); formal analysis (supporting); methodology (equal); project administration (equal); supervision (lead); writing – review and editing (equal).

## CONFLICT OF INTEREST

All authors declare that they have no conflicts of interest.

## ETHICS STATEMENT

This study conforms to the recognized standards according to the Declaration of Helsinki. The Regional Ethics Committee of South East Norway approved this study (REK Sørøst). Clinical trial number is 2012/1141. Written consent was obtained from all the participants.

## Data Availability

The data that support the findings of this study are available from the corresponding author (KH) upon reasonable request.
